# Supplementary material for: Interleukin-33 Induces Neutrophil Extracellular Trap (NET) Formation and Macrophage Necroptosis via Enhancing Oxidative Stress and Secretion of Proatherogenic Factors in Advanced Atherosclerosis
Source: Antioxidants (Basel). 2022 Nov 26;11(12):2343. doi: 10.3390/antiox11122343 (PMC9774908; doi:10.3390/antiox11122343)
Supplement: Supplementary file 1 [file antioxidants-11-02343-s001.zip › antioxidants-2031046-supplementary.pdf]

Table S1: Effect size analysis of all the test parameters reported in the study

Test parameters	aAT<br>Mean ± SD	Control<br>Mean ± SD	Cohen's d (Effect size)<br>(95% confidence Interval lower, Upper)
**Oxidative Stress markers (Calorimetric method based assay)**			
8-OHdG<br>(ng/ml)	20.467± 12.294	8.725± 7.255	1.163<br>(0.744, 1.577)
MDA<br>(nmol/ml)	8.376± 4.938	5.815± 2.776	0.640<br>(0.244, 1.032)
GSH-GSSG ratio	7.462±4.335	4.884± 2.787	0.707<br>(0.308, 1.108)
SOD<br>(Units/mg)	9.469± 6.802	16.742± 11.031	-0.793<br>(-1.191, -0.392)
CATALASE<br>(Units/mg)	16.584± 14.987	41.531± 37.051	-0.883<br>(-1.283, -0.477)
**Atherogenic factors in treated macrophages (Transcript, $2^{-\Delta Ct}$)**			
IFN-γ	0.0018± 0.0024	0.0004± 0.0003	0.833<br>(0.4297, 1.2316)
TNFα	0.0074±0 .0180	0.0013±0.0022	0.476<br>(0.0857, 0.8654)
IL-1β	0.0051±0.0114	0.0016± .0015	0.438<br>(0.0481, 0.8262)
IL-6	0.0128± 0.0215	0.0027± 0.0026	0.622<br>(0.2657, 1.0556)
IL-10	0.0022± 0.0024	0.0031± 0.0039	-0.284<br>(-0.6698, 0.1028)
IL-12B	0.0006±0.0006	0.0004± 0.0006	0.298<br>(-0.0897, 0.6833)
IL-18	0.0075± 0.0162	0.0044± 0.0098	0.239<br>(-0.1468, 0.6247)
IL-23	0.0089± 0.0133	0.0029± 0.0038	0.600<br>(0.2059, 0.9921)
IL-33	0.0020± 0.0019	0.0012± 0.0015	0.463<br>(0.0726, 0.8518)
MCP1	0.0013± 0.0019	0.0005± 0.0009	0.513<br>(0.1212, 0.9028)
GMCSF	0.0019± 0.0038	0.0009± 0.0015	0.346<br>(-0.0426, 0.7320)
**Expression in neutrophils (Transcript, $2^{-\Delta Ct}$)**			
MPO	0.0022± 0.0047	0.0003± 0.0006	0.569<br>(0.1761, 0.9605)
IL-33R	0.0053± 0.0095	.0014± .0025	0.562<br>(.1687, .9528)
**Necroptosis markers in treated macrophages (Transcript, $2^{-\Delta Ct}$)**			
RIPK1	0.0021± 0.0039	0.0013± 0.0021	0.239<br>(-0.1466, 0.6249)
RIPK3	0.0037± 0.0082	0.0025± 0.0052	0.186<br>(-0.2001, 0.5703)
MLKL	0.0040±0.0025	0.0049± 0.0092	-0.137<br>(-0.5218, 0.2478)
**Inflammasomes (Transcript, $2^{-\Delta Ct}$)**			

AIM2	0.005±0.0093	0.0034±0.0039	0.242<br>(-0.1437, 0.6278)
NLRP1	0.0032±0.0032	0.0021±0.0023	0.389<br>(0.00023, 0.7763)
NLRP3	0.0095±.0181	0.0041±0.0075	0.390<br>(0.0014, 0.7775)
NLRC4	0.0161±0.0256	0.0039±0.0049	0.657<br>(0.2609, 1.0504)
**TIMPs and MMPs (Transcript, $2^{-\Delta Ct}$)**			
TIMP1	0.0046± 0.0090	0.0151± 0.0255	-0.547<br>(-0.9377, -0.1544)
TIMP2	0.0039± 0.0057	0.0075± 0.0120	-0.371<br>(-0.7578, 0.0175)
TIMP3	0.0012± 0.0019	0.0028± 0.0042	-0.495<br>(-0.8843, -0.1037)
TIMP4	0.0038± 0.0055	0.0076± 0.0141	-0.348<br>(-0.7348, 0.0398)
MMP-1	0.0017± 0.0024	0.0013± 0.0018	0.232<br>(-.1541, .6173)
MMP-2	0.0019± 0.0027	0.0013± 0.0024	0.233<br>(-0.1530, 0.6183)
MMP-3	0.0081± 0.0180	0.0017± 0.0028	0.491<br>(0.1004, 0.8809)
MMP-7	0.0032±0.0078	0.0017± 0.0030	0.241<br>(-0.1445, 0.6270)
MMP-9	0.0012± 0.0023	0.0006± 0.0015	0.330<br>(-0.0578, 0.7162)
MMP-12	0.0069± 0.0273	0.0022±0.0057	0.234<br>(-0.1519, 0.6195)
MMP-13	0.0046± 0.0134	0.0009± 0.0019	0.383<br>(-0.0049, .7710)
MMP-14	0.0019± 0.0022	0.0016± 0.0021	0.135<br>(-0.2490, 0.5206)
**ELISA based protein quantification data**			
MPO (PLAQUE)<br>(pg/ml)	283.4 ± 169.7	--	--
IL-33R (PLAQUE)<br>(pg/ml)	76.08 ± 50.29	--	--
RIPK-1<br>(ng/ml)	0.492 ± 0.384	0.849 ± 0.575	0.730<br>(.3317, 1.1261)
RIPK-3<br>(ng/ml)	1.511± 0.955	1.040± 0.551	0.604<br>(.2089, .9953)
MLKL<br>(ng/ml)	2.597± 2.10	1.822± 0.910	0.701<br>(.3030, 1.0954)
